# Applying the theoretical domains framework and behavior change wheel to inform interventions for food and food-related waste audits in hospital foodservices

**DOI:** 10.3389/fnut.2023.1204980

**Published:** 2023-08-15

**Authors:** Nathan Cook, Jorja Collins, Judi Porter, Denise Goodwin

**Affiliations:** ^1^Department of Nutrition, Dietetics and Food, Monash University, Notting Hill, VIC, Australia; ^2^Eastern Health, Box Hill, VIC, Australia; ^3^School of Exercise and Nutrition Sciences, Institute for Physical Activity and Nutrition, Deakin University, Geelong, VIC, Australia; ^4^BehaviourWorks Australia, Monash University, Clayton, VIC, Australia; ^5^Monash Sustainable Development Institute, Monash University, Clayton, VIC, Australia

**Keywords:** food waste, audit, foodservice, hospital, behavior change

## Abstract

**Background:**

Completing aggregate food and food-related waste audits in hospital foodservices is an intense practice, however they can demonstrate problem areas that require attention to reduce waste. Identifying interventions to facilitate and improve the implementation of these audits can be guided by behavior change science. The aims of this study were to use behavior change theories and frameworks to (1) describe the drivers of behavior to complete food and food-related waste audits and (2) identify possible interventions that support the implementation and uptake of these audits.

**Methods:**

Purposive sampling was used to recruit participants from hospitals in Victoria, Australia who worked in their foodservice system. Semi-structured interviews sought knowledge of participant’s perceived barriers and enablers to completing food and food-related waste audits. Deductive analysis using the Theoretical Domains Framework (TDF) and Capability Opportunity Motivation Behavior theory (COM-B) identified dominant drivers of behavior. TDF domains were then matched to their corresponding intervention functions according to the Behavior Change Wheel framework (BCW) to identify relevant strategies that may support audit implementation.

**Results:**

Data from 20 interviews found the dominant COM-B constructs (TDF domains) were psychological capability (knowledge, skills), physical opportunity (environmental context and resources), and reflective motivation (social/professional role and identity, beliefs about capabilities). These dominant domains come from narratives that participants shared about foodservice staffs’ lack of knowledge, labor, time, and the hospital avoiding responsibility for audit completion. Corresponding intervention functions that could have the most potential for implementing waste audits were education, training, environmental restructuring, modeling, and enablement. Participants’ shared perspectives of audit enablers resembled these: for example, obtaining staff buy-in, reinforcing behavior through incentives and installing an audit champion.

**Conclusion:**

To transition toward regular food and food-related waste auditing practices in hospital foodservices these findings may help identify practice and policy change that delivers standardized auditing activities to encourage long term behavior change. Interventions to support audit completion should address each behavioral construct and relevant domain, as individual hospital sites will experience unique contextual factors and expectations influencing audit outcomes. A co-design process that includes staff and stakeholders of hospital foodservices is recommended to enable engagement and practical solutions to audit implementation.

## 1. Introduction

Food waste definitions differ in what they consider, such as the edible (e.g., apple skin) or inedible (e.g., apple core) material of food that is otherwise discarded, lost or consumed by pests along the food supply chain (1). Food-related waste describes the packaging of these foods to move them safely through the food supply chain. For the purpose of this study, the term food waste will incorporate both the edible and inedible portion of food as described above and defined by Garcia-Garcia et al. (2). Collectively, although food waste is lower at the consumption stage of the food supply chain when compared to other stages it has the highest carbon footprint (1, 3). This is due to the accumulation of resources to move food from “field to fork” or from the agricultural production stage through to households, restaurants, and large organizations containing a foodservice, such as hospitals, schools, and aged care. For hospitals in particular, reducing food waste at the consumption end is an important milestone for moving toward and contributing to sustainable healthcare (4). What makes this difficult, however, is that food waste at this stage of the food supply chain is in part caused by or related to human behavior (5) such as over ordering by patients (6), preparation mistakes by cooks (7), and incorrect separation of food into waste categories by staff that is governed by hospital food safety regulations (8).

Evidence has demonstrated that up to 50% of all hospital waste is comprised of food waste including food scraps from meal preparation, surplus food on the plating line, and plate waste returned to the kitchen after service (9, 10). The reasons for this waste have been described previously, and include patient interest in food and their appetite, the quality and quantity of food, and the design of the foodservice system (6). Regardless of where food waste occurs within this system, this is a significant waste of resources such as money and labor which includes time spent on producing food that is ultimately wasted. The current scenario is not only slowing the wider healthcare systems’ realization of sustainable practices, but also failing the global attempt to reach Sustainable Development Goal (SDG) 12.3, which aims to reduce the environmental impacts associated with food waste through reduction initiatives that by 2030, halve *per capita* global food waste (11). This sub-goal is one of many from the 17 primary objectives designed by the United Nations in 2015 to promote peace and prosperity for people and the planet, now and into the future (12).

One strategy suggested to combat this problem by international roadmaps and commitments to SDG 12.3 (12–16) is to systematically measure aggregate food waste on a regular basis. This helps to understand baseline levels of waste, set goals for reduction and to monitor waste quantities regularly and consistently over time. An evidence-based food waste audit tool has been developed to facilitate hospital foodservices’ decision making when designing a waste audit (9). The process involves planning logistics, obtaining necessary equipment such as scales to measure waste, training the workforce, choosing what type of waste to measure, where and how to measure it, and analyzing the results. However, it can be challenging for hospital foodservice staff to complete additional tasks due to the nature of the foodservice environment (e.g., limited time) (17, 18). Furthermore, minimal staff training, data collection problems, audit method feasibility (9), reduced staffing resources, and availability of space are additional challenges reported to influence waste audits in hospital foodservices (18).

Understanding the drivers for behaviors of the hospital foodservice workforce to effectively complete food and food-related waste audits can inform their implementation (19). Yet individual human behaviors are complex, multilevel, multipronged and take place in different contexts and settings (20). Often behaviors, such as completing a food and food-related waste audit, are not singular behaviors with singular solutions, but require deeper understanding to promote change. As hospital foodservice responsibilities are divided across a diverse group of staff members, to achieve the desired behavior of food and food-related waste audit completion there are potentially many behaviors that could be defined and addressed (using an outline such as Actor Action Context Target Time) (21). For example, a foodservice staff member weighing food correctly, or a foodservice manager scheduling a team meeting, are two behaviors that are linked in a chain of many more behaviors. Instead of promoting common strategies that can be ineffective, such as only providing information, and trying to predict behavior (20) by assuming people always behave rationally, the application of behavior change theories and frameworks may support desired change.

Behavior change theories are structured statements of hypothesized processes that explain the concept of behavior change (22), whereas behavior change frameworks provide systematic methods which can support intervention design to drive change, and incorporate an understanding of behavior (23). The Behavior Change Wheel (BCW) is an overarching framework of behavior derived from 19 preceding behavior change frameworks (22). Within the most inner layer of the wheel are the essential interacting theoretical components of Capability (physical or psychological), Opportunity (physical or social), and Motivation (emotional or reflective) that drive behavior (the COM-B model). In the middle layer of the BCW are nine intervention functions which are strategies that can target behavior change. The most outer layer of the BCW is comprised of seven policy categories which could enable these intervention functions to occur. The BCW is a framework that has supported researchers’ theoretical understanding of behavior to decide what change is required to modify current behaviors, and inform clinical practice changes in various settings (24).

The Theoretical Domains Framework (TDF) is a framework synthesized from 33 theories and 128 constructs for use in implementation research, with the updated and validated version including 14 domains and 84 constructs (25). The TDF is associated with the theory based COM-B system whereby Capability, Opportunity, and Motivation are subdivided further into the 14 domains of the TDF (24). The TDF has been widely used in healthcare research to explore what influences healthcare professionals’ behavior in relation to; the use of evidenced-based guidelines, the barriers and enablers to intervention uptake, to investigate intervention design, and conduct process evaluations (22). Together, this one theory (COM-B) and two frameworks overlap (BCW, TDF) and can be used in a combined approach to sequentially map behavioral constructs from the COM-B and TDF to corresponding intervention functions and policy categories from the BCW that may be effective in creating behavior change (24). The current study uses these theories and frameworks as a method to investigate factors influencing the completion of food and food-related waste audits in hospital foodservices.

Aggregate food and food-related waste audits in hospital foodservices are those that identify, collect and measure food (e.g., patient left overs) and food-related waste (e.g., yoghurt sachet) produced by the foodservice operation during the preparation, plating, and return of food intended for patient consumption. Currently, there is minimal evidence (18, 19, 26) regarding the barriers and enabling factors that foodservice staff experience in relation to completing food and food-related waste audits, and what actions would support implementation within the hospital setting. To the knowledge of the research team, evidenced based behavior change theories and frameworks have also not been used to support the implementation of food and food-related waste audits in hospital foodservices (27). Therefore, the aims of this study were to use behavior change theories and frameworks to (1) describe the drivers of behavior to complete food and food-related waste audits and (2) identify possible interventions that support the implementation and uptake of these audits.

## 2. Methods

Ethics approval was obtained from the Monash University Research Ethics Committee (Project ID: 28908) and reporting followed the consolidated criteria for reporting qualitative research (COREQ) guidelines (28).

### 2.1. Design

Generic qualitative enquiry was used to investigate the research questions through the completion of individual semi-structured interviews, allowing participants to share their unique perspectives and experiences (29). The theoretical positioning underlining this study was interpretivism (30) as the research questions aimed to explore participants’ perspectives on the topic of interest. Interpretivism is underpinned by a relativist ontology and subjectivist epistemology whereby participants’ individual realities are derived from previous experiences and the meaning they give them. Therefore, there are multiple realities which can be explored and interpreted when drawn out from discussions between participants and the researcher.

### 2.2. Participants and recruitment

This study purposefully recruited participants who worked in or managed the foodservice at a public hospital in Victoria, Australia and had knowledge of their hospital foodservice operations.

From a list of public hospitals in Victoria (31), 10 hospitals were selected using maximum variation sampling (32) and telephoned per fortnight to acquire the operational manager’s email address, who were then contacted to inform them of the study details and requirements. Consistent with ethics requirements, a signed letter of support was sought from the organization and written informed consent was sought from individuals participating in an interview. To extend the reach of this study, snowball sampling was used, whereby after the formal interview ended, participants were requested to reach out to any colleagues who may meet the eligibility criteria and ask them to contact the research team (33).

To understand the sample size required, the concept of evidentiary adequacy was used (34). This considers that data collected contain an adequate amount and variety of evidence that could be interpreted, and is disconfirming and discrepant. These conditions were deemed satisfactory by the research team before recruitment ceased.

### 2.3. Data collection

An interview protocol (19) was designed and piloted with hospital foodservice workers and researchers (*n* = 9), prior to being edited and used in interviews. In addition, an experienced qualitative researcher from the team reviewed the first transcript and provided feedback to the interviewer before continuing with data collection. Six questions were focused on participants’ perceptions of the barriers and enablers toward food and food-related waste auditing in hospital foodservices, and reflections on a published consensus tool (9) that guides users on how to complete food and food-related waste audits. This protocol in full detail is available within a separate publication reporting on this data-set (19). The TDF was not consulted to design the interview protocol.

Interviews were conducted between August and November 2021, by the primary researcher who was a higher degree research student and an Accredited Practicing Dietitian. The video communications program Zoom (Version 5.5, Zoom video communications, California) was utilized and interviews were recorded. Participant and organization information collected included age, gender, years in current position, previous work experience in foodservice, hospital size, and foodservice model. No interviews were repeated and transcripts were not provided to participants for review (member checking) to decrease participant burden as interviews occurred during the height of the COVID-19 pandemic. Additionally, field notes were not taken during data collection so the interviewer could dedicate their attention to the participant. Therefore, to allow reflexivity, every fortnight during data collection three members from the research team, including the interviewer, met and discussed the data and its meaning (peer debriefing) (35).

### 2.4. Data analysis

Descriptive statistics were used to report participant and hospital demographic data. The artificial intelligence software Otter.ai (Version 2.1.52, Otter.ai, California) transcribed interview recordings, which were then reviewed and edited for accuracy by listening to the audio and reading the transcript. Inductive thematic analysis was conducted in NVivo (NVivo, QSR International, Victoria) by the primary researcher to generate codes from the data (36). At the same time, transcripts were deductively analyzed by overlapping these generated codes with appropriate TDF domains as secondary codes (22). The final stage of coding matched TDF domains to their overarching COM-B construct (24). To ensure data were coded appropriately and assigned to a suitable TDF domain, 10% of coded transcripts were reviewed by one other experienced qualitative and behavior change researcher; discrepancies were discussed and amended for consensus.

To identify the most relevant TDF domains to inform interventions for the research area under investigation, authors (22, 37, 38) have previously applied three criteria: relatively high frequency of specific beliefs and/or themes, presence of conflicting beliefs, and perceived evidence of strong beliefs that may affect the target behavior. Therefore, in this study, the frequency of codes (number of times reported), presence of conflicting codes (evidence of barriers and enablers in the same domain), and perceived evidence of strong codes (how strong these codes are thought to influence behavior by the primary researcher) were used to demonstrate relevant TDF domains. The relevant domains were then matched to their identified intervention functions using a domain-intervention linking matrix and following steps 1–5 of 8 from the BCW method (24).

### 2.5. Relevant intervention functions and dominant COM-B constructs (TDF domains)

**Figure 1:**
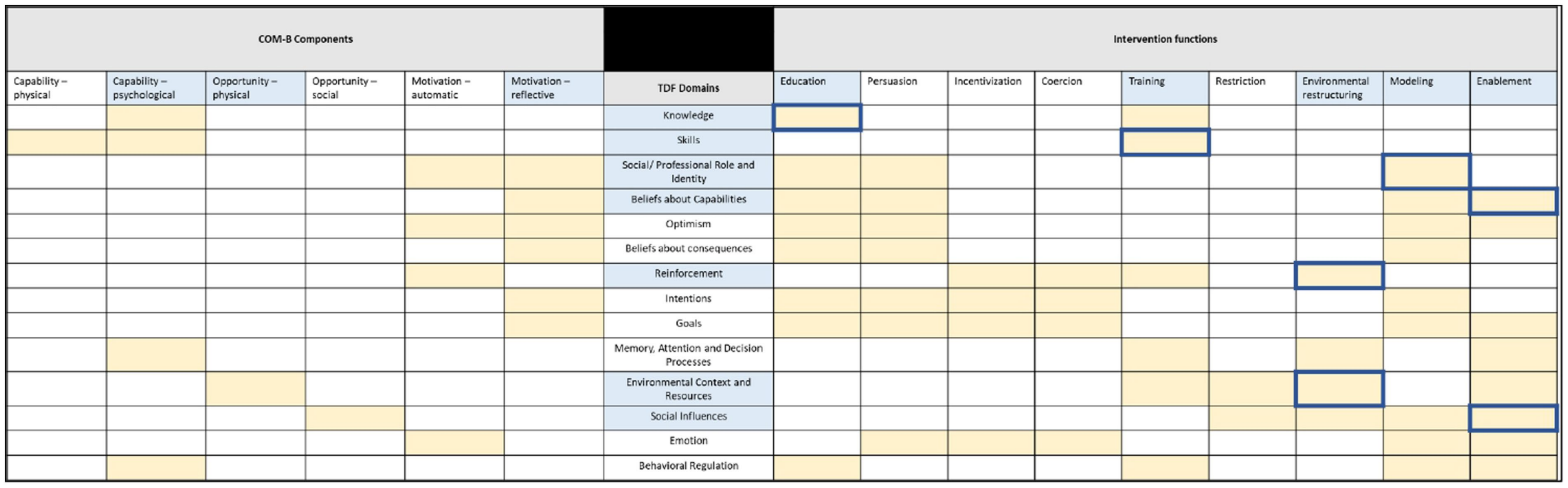
The relationships between COM-B components, the TDF domains, and Intervention Functions. Light blue squares are the dominant parts of each framework based on the results from this study; yellow squares are identified relationships between the frameworks from Michie et al. (1); dark blue border squares are the intervention functions that support behavior change in hospital food and food-related waste audits based on participant responses.

The COM-B theory and TDF behavior change framework were used to identify factors influencing food and food-related waste audits occurring in hospital foodservices, and the aligning behavior change interventions that are best suited to promote waste audits to occur (Figure 1). All COM-B components were contained within the results, with the dominant components being psychological capability, physical opportunity, and reflective motivation. The associated TDF domains were environmental context and resources, social/professional role and identity, knowledge, reinforcement, social influences, beliefs about capabilities, and skills. Domains were deemed relevant as evidenced by their coded frequency and examples of participant responses are detailed in Table 1.

**Table 1:** Participant reported barriers and enablers, their coded themes and relationship to the applied behavior frameworks.

TDF domain	COM-B component	Coded theme	Example quote (participant number)	Barrier ✖ or enabler ✔
Environmental context and resources	Physical opportunity	Time	Yeah, the time to actually plan it, and who would have that responsibility, and then the actual time of staff to do it. They are already I think, in most hospitals, they are pretty pushed to get jobs done within their rostering and so to get anything that changes their normal practice is seen as a barrier to doing it and particularly if it adds time (P5).	✖
		Labor	I suppose it would be a great idea, if we could have an external auditor come in once every so often, just like we get in for calibration of our monitors, you know, once every six months or whatever, and doing an audit. Even if it was just a snap shot, so if they did it the same time each year we might get some continuity there (P8).	✔
Social/professional role and identity	Reflective motivation	Staff population	Also, I was going to say that basically peoples’ willingness to do what they have been asked to do, I think there is some real pushback around that. And that is it. That is a general issue that we have within our staff base with some individuals, and I think you will find that in most organizations with this particular cohort (P2).	✖
		Staff buy-in	And so, it is just talking to these people, talking to all the staff that are involved, and getting them to buy into it, they need to buy into it (P9).	✔
Knowledge	Psychological capability	Staff population	You [interviewer] may think that it is actually very easy. But like I said, different staff have different knowledge, so it is actually quite hard. And the fact you must remember is that they [foodservice staff] are paid to do only certain roles and certain jobs… (P10).	✖
		Education	I think it all just generally falls back to training. And it needs to be robust training so that everyone knows exactly what needs to be done (P1).	✔
Reinforcement	Automatic motivation	Staff population	I am the Chief Sustainability Officer, I can say *x*, *y*, and *z* and put in place [and audit] all decided, but there is 9,000 people in this organization, if they are not engaged, it’s not going to work (P19).	✖
		Staff buy-in	So, I think if we said to them, look we are going to reduce our food waste and were going to get a new dishwasher, they would be like, oh, thank God that makes our job so easy, because our dishwasher is so old, so yeah, things like that would help (P17).	✔
Social influences	Social opportunity	Change	I mean, there is a ways and means of doing it, but we have an older cohort of staff who can be quite routine in their habits. So, trying to change those habits as well put processes in place, I think, at the moment, they are [the staff] quite resistant to additional tasks being laid upon them (P2).	✖
		External pressure	I think it’s got to be led from the top, whether it’s the top within the health service, or whether its governed by an EPA [environmental protection agency] or something like that just to have some reporting back to it, to a higher body (P12).	✔
Beliefs about capabilities	Reflective motivation	Staff population	I think it is probably the staffs time or their perception of their capacity to do that within their working day. An whose responsibility that would be so that is the first one because we get pushback around time on other processes within the hospital kitchens that we are trying to work toward (P2).	✖
		Staff buy-in	I think it is about not coming to the foodservice staff with an already pre-written plan of what exactly needs to be done and how it needs to be done. Because actually, foodservice staff have so much knowledge of the kitchen and how it works. They are in there every day. So, I think that going to them and framing it as more of a workshop like, okay, this is what we are doing, this is why we are doing it… Now how do you think you can make it work? and having some ideas already, but really giving them some ownership and letting them guide the process (P13).	✔
Skills	Physical and psychological capability	Staff population	So, we are just trying to make sure that everyone is not putting in incorrect data, because then everything is going to be wrong. And there is no point if the data are incorrect, then there is no point in doing the whole exercise (P9).	✖
		Education	…like is there training that they can do whether within the organization or external to the organization that actually builds those skills because, you know we [dietitians] learn those skills from university and from in school and things like that, but yeah, they have not… (P17).	✔

**Figure 2:**
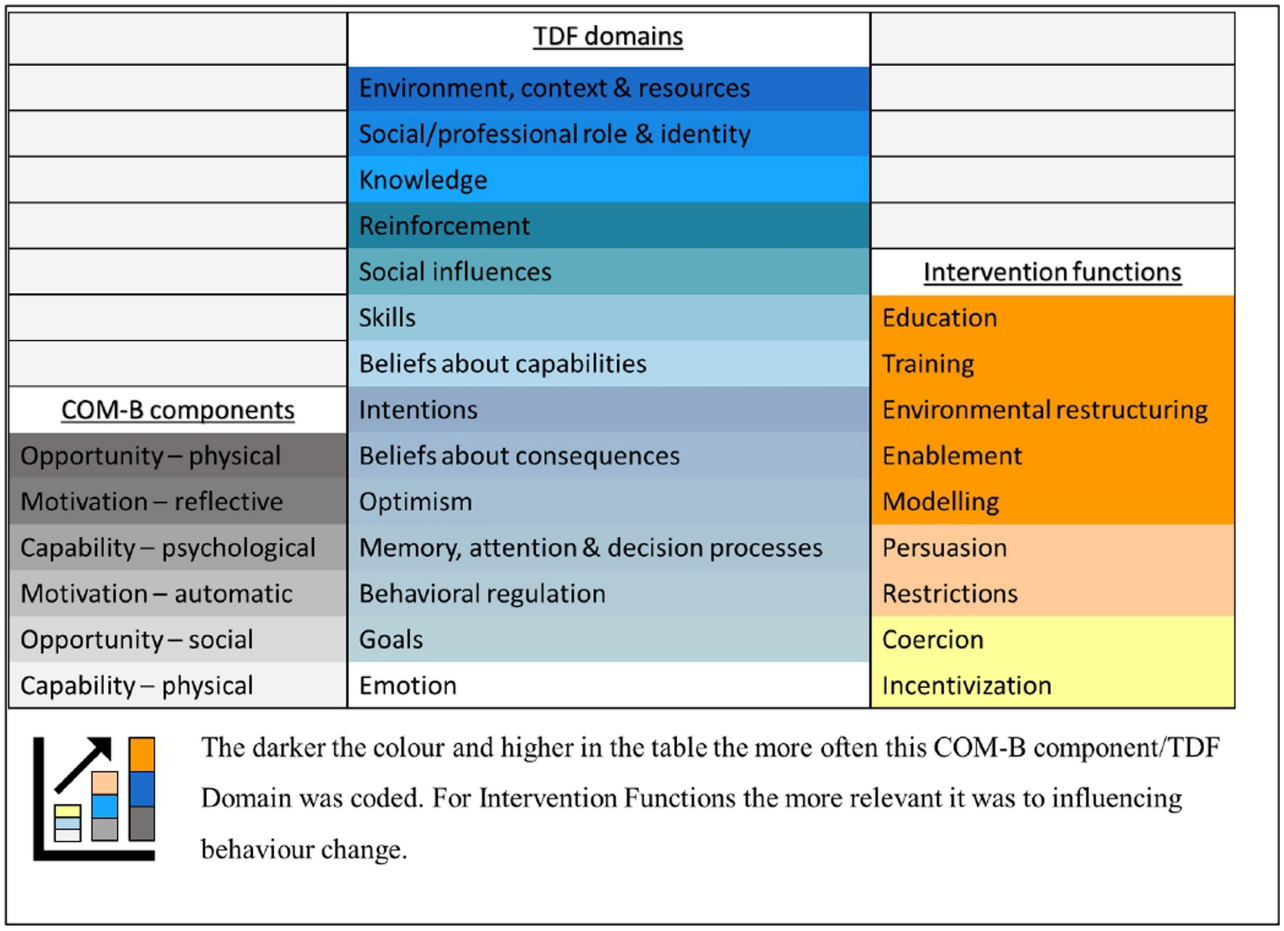
COM-B components/TDF domains and relevance of corresponding intervention functions represented as a heat map.

When the dominant TDF domains were mapped to the intervention functions matrix, the most common intervention functions to target a change in behavior were: education, training, environmental restructuring, modeling, and enablement (Figure 2). These align with enablers participants suggested to support the completion of food and food-related waste audits in hospital foodservices, for example; education sessions (education), upskilling the workforce with training (training), purchasing electronic data collection software (environmental restructuring), scheduling trial audits (modeling), and designing the audit to be easy (enablement) (24).

## 3. Results

### 3.1. Participants

During the recruitment period, 70 hospitals were contacted. The majority of hospitals either did not respond (75%) or did not accept study invitations for reasons including: not sharing participant contact details (6%) and competing priorities (4%). The final sample included 20 participants from nine health services (15%) (26). One additional participant who completed their interview withdrew their data for unknown reasons and was not included in analysis. Five participants described completing previous food waste audits (food only) for surplus unserved waste on the plating line or patient plate waste.

More than half of participants were female (60%), and the mean age of all participants was 44 years. Participants came from different hospitality backgrounds both inside and outside of hospital foodservices, and had been working in their current role for at least 2-months. The most common position was foodservice dietitian (*n* = 4), followed by hotel services coordinator (*n* = 2) and project coordinator (*n* = 2). There were a number of manager/supervisor roles including: support services (*n* = 1), foodservice (*n* = 1), group management services (*n* = 1), catering (*n* = 1), facilities services (*n* = 1), food safety (*n* = 1), dietetics (*n* = 1), and sustainability (*n* = 1). Additionally, there were one each of a store person, head chef, catering dietitian, and sustainable food systems dietitian. There was crossover between reported job titles and responsibilities including management of the department and supervision of staff, menu development, project initiation and food preparation, plating and service. The hospital size ranged from 18 to 600 beds, and the most common foodservice type was cook chill. Interview length ranged between 50 and 94 minutes (mean time 64 min).

The following results describe the behavioral drivers to enable food and food-related waste audits in hospital foodservices reported by participants, and the intervention functions that could support audits moving forward as interpreted using the COM-B, TDF, and BCW.

### 3.2. Education

#### 3.2.1. Capability: psychological (knowledge)

Participants reported that individuals and groups who could be involved in a food and food-related waste audit may not have the knowledge on how to execute one and why. However, educating a large variable workforce was perceived as being complex, and would take time and repetition to include the entire workforce. The appropriate intervention function to target the knowledge domain from the TDF is education. Educating the workforce was a continuous message from participants to ensure foodservice staff had the appropriate knowledge of why they were being asked to complete a food and food-related waste audit; the benefits to them and the hospital, and how it effects their day-to-day operations. Developing paper based or online education material and launching this at meetings was recommended.

One participant (P13, Foodservice dietitian) alluded to a gap at their site where education had not occurred prior to previous audits. Further, other participants who had completed a food waste audit (food only) explained how even though they delivered some education for staff, there were still errors in visual estimation of different food items. Constant communication through multiple channels (e.g., internal corporate communication strategies) was suggested as a major component to delivering effective education to staff, and was highlighted by one participant (P16, Catering manager) as a useful approach to demonstrate to the audit team the value of their efforts.

### 3.3. Training

#### 3.3.1. Capability: physical and psychological (skills)

A wide range of skills were perceived to be necessary to apply the food and food-related waste audit consensus tool and complete an audit including: planning the most suitable audit method, procuring resources for use, skills in data collection methods (e.g., food waste weighing), and computer skills for data entry. However, one participant (P17, Foodservice dietitian) highlighted how these skills are not essential for those staff expected to complete the audits in their current roles. As a result, the accuracy of audit data was a concern from some participants, and may lead to an unexpected time commitment on their behalf for the checking of collected audit data. The dedicated intervention function to supporting the physical and psychological skills domain from the TDF is training.

Upskilling audit staff through internal or external training modules focused on audit methods and computer skills were recommended by participants to support food and food-related waste audit completion. To ensure skills were adequate, different participants suggested; practicing audit specific skills, running workshops, documenting correct procedures, and developing assessment items to work through data collection issues. Additionally, training was encouraged to be frequent, mandatory, clearly detailed, and delivered to the audit staffs’ level of understanding. Participants also recommended that audit processes that were fast and easy may reduce the skill level required to complete audits, which could perhaps entice staff from other departments who would not typically be involved to be included (e.g., hotel services, nursing).

### 3.4. Environmental restructuring

#### 3.4.1. Opportunity: physical (environmental context and resources)

The foodservice setting was labeled as a time driven department with strict schedules and deadlines that governed staff movement throughout the day. Food and food-related waste audits were perceived to disrupt foodservice operations due to the time required to plan audit necessities such as resource needs (e.g., physical or digital data collection tool, equipment to measure and store waste, and audit method training materials), the amount of additional work they create and the extra manpower required to collect, separate, weigh, and discard measured food waste. These activities were perceived to take time away from standard business activities. One participant (P17, Foodservice dietitian) reported their foodservice department experienced high levels of staff sick leave resulting in reallocation of tasks and this would disrupt the workflow during an audit. Environmental restructuring which involves changing the physical or social context surrounding a behavior is one intervention function suggested to support individual’s physical opportunity to complete tasks.

Different strategies were suggested by participants to overcome the two major hurdles perceived to inhibit food and food-related waste audits: labor and equipment. These included having a part-time or casual staff bank to call upon when needed, introducing a dedicated shift to audit waste, collaborating with staff from other departments, utilizing university work integrated learning students, hiring externally qualified auditors, using devices and software to support data collection, and having the appropriate equipment and place to store it. Participants felt that the essential equipment required to support a smooth audit process were items such as scales, gloves, cameras, bins, buckets, and collection trolleys. One participant (P13, Foodservice dietitian) explained how including both dietitians as well as foodservice managers supported their food waste audit (food only) as it reduced reliance on only one set of staff.

#### 3.4.2. Motivation: automatic (reinforcement)

Reinforcement is another TDF domain which is influenced by the intervention function of environmental restructuring as a strategy that may support motivation to complete tasks. However, participants commented that foodservice staffs’ personal beliefs about audits and their environmental benefits and the lack of policy mandating hospital foodservice food and food-related waste audits were barriers to reinforcing their importance. Participants also reported that staff would view them as “*just another manager introducing more work*” or increasing work demands “*not included in their job descriptions.*”

To reinforce audit behaviors participants recalled strategies they had used to change the environment in the past including staff competitions or visual progress boards and offering staff incentives in exchange for their efforts such as extra money toward a work function or to upgrade equipment that would increase efficiency. One participant said having staff who cared about food waste supported their previous audit’s success (P17, Foodservice dietitian). Additionally, continually checking in on staff and reminding them of audit methods during the audit was proposed by some to perhaps reinforce accurate practice. Governance of foodservice operations in the form of policy, guidelines, or standards was also recommended by participants to support audits occurring in practice.

### 3.5. Modeling

#### 3.5.1. Motivation: reflective (social/professional role and identity)

Modeling desired behaviors to set an example for people to aspire to or imitate is the intervention function that targets the TDF domain of social/professional role and identity. Participants described that foodservice workers’ did not see food and food-related waste audits as part of their role, and staff reported questioning why it was their responsibility. Participants believed foodservice staff had a low willingness to follow new directives and low organizational support from an executive level decreased interest. Hospitals foodservices as a department were labeled by one participant (P17, Foodservice dietitian) as perhaps having a lower work satisfaction culture, which could explain why foodservice staff were reluctant to complete food and food-related waste audits.

A common enabler reported by participants included the process of involving and empowering foodservice staff in food and food-related waste audits to ensure their “buy in.” Giving the workforce ownership of the project may improve their commitment toward completing a food and food-related waste audit and help establish a group identity associated with this task. One strategy suggested to achieve this was instigating a “champion” such as the foodservice manager, as messages are often received clearer by foodservice staff from internal supervisors compared to executive administration staff. Some sites had an active sustainability working group that met regularly and participants suggested this group could adopt a food and food-related waste audit project to model organizational responsibility to the wider foodservice team.

### 3.6. Enablement

#### 3.6.1. Opportunity: social (social influences)

Enablement as an intervention function is described as supporting capability and opportunity to engage in a behavior beyond other strategies of education, training, and environmental restructuring. Foodservice staff were described as being resistant to change, which hindered food and food-related waste audits. One participant (P3, Special projects coordinator) described how they believe some foodservice staff may seek the easiest option rather than the correct option in regards to work tasks, and they were worried this individual agency may lead to problems such as improper sorting of waste. Additionally, one participant (P1, Support services manager) explained how foodservice staff tend to be part of industry unions, and therefore have working agreements that can make incorporating other tasks to their roles problematic.

Teamwork was suggested by participants to be a strategy that may help influence audit completion. A Chief Sustainability Officer (P19) commented that food and food-related waste audits require a team effort due to it being a small project inside a large organization. Top down approaches (i.e., mandated by the senior management) were not recommended by this same participant as they highlighted that as a leader, they need to understand specific stakeholder barriers to audit completion within the team so they can develop solutions that can enable individuals to engage and provide opportunity for staff involvement. Further, a Support services manager (P11) reported that working with other hospitals to find solutions to complex problems was a successful strategy in the past.

#### 3.6.2. Motivation: reflective (beliefs about capabilities)

Many intervention functions can affect the TDF domain of beliefs about capabilities, however enablement was most aligned to the behaviors described by participants in this study. University nutrition students on work integrated learning placements were commonly referred to by participants as having a higher capability than foodservice staff to undertake audits based on their topic knowledge and availability. Examples were given of students completing audits as a placement project, with the foodservice workforce then considering their suggestions for operation changes to reduce food waste.

A Foodservice dietitian (P17) expressed that foodservice staff do not realize their importance in the hospital’s system. Empowering staff who are concerned about food waste to realize their ability to change the outcomes of the problem was described as an enabler. A Foodservice dietitian (P13) reported that when completing a food waste audit (food only), consultation with their foodservice staff on possible barriers and discussion of the audit design supported their motivation and willingness to be involved. Some participants recommended completing a trial audit with foodservice staff to develop their familiarity with the audit method and improve their perceived self-confidence before executing a real audit. Other suggestions included separate data collection over meal times and teaching staff to use electronic data collection tools, as these activities may enable belief in their own capabilities.

## 4. Discussion

The aims of this study were to use behavior change theories and frameworks to (1) describe the drivers of behavior to complete food and food-related waste audits and (2) identify possible interventions that support the implementation and uptake of these audits. The application of the BCW, TDF, and COM-B to participant interview data highlighted the key behavioral drivers which support a food and food-related waste audit and helped interpret the interventions which can promote these behaviors. There was a clear dominance from the COM-B (22) components; psychological capability, physical opportunity, and reflective motivation, which coincide with the TDF (25) domains of knowledge, environmental context and resources, and social/professional role. These dominant domains come from key common narratives from participants about foodservice staffs’ lack of knowledge about food and food-related waste audits, labor, time, and equipment limitations, and the hospital and its staff avoiding responsibility for food and food-related waste audits. Participants suggested enablers to counteract these barriers, which are also reported elsewhere, including; trialing food and food-related waste audits to familiarize staff with the audit procedure (39), developing educational resources for staff viewing (39), measuring waste with appropriate weighing scales (40–42), and training staff on appropriate weighing methods before audits begin (39, 41, 43). While previous research indicates foodservice staff already acknowledge what is required to complete a waste audit, these studies haves not used behavioral science theory to identify if the chosen interventions were the most appropriate strategies to facilitate long lasting food and food-related waste auditing behaviors in their hospitals.

Building an implementation strategy using the intervention functions highlighted in the current study (education, training, environmental restructuring, modeling, and enablement) may support future audit uptake in hospital foodservices. Tailoring interventions toward subgroups in their specific contexts (e.g., chefs) rather than targeting a large population of individuals (e.g., the entire foodservice) has been suggested (24). A systems thinking approach could be applied to identify cause and effect relationships of behaviors at different subgroups of the larger system. Examples of this include the micro, meso, macro (44) or individual, social, material (45) approaches that represent the different levels suggested by participants in this study which determine the completion of food and food-related waste audits; foodservice staff member (micro), foodservice manager (meso), and hospital organization (macro). Intervention functions could be focused on specific target behaviors completed by these groups in the system, as specific stakeholders (e.g., executives vs. dietitians) have different influences on practice.

The principles of co-design (collaborative design) (46) have been previously used in healthcare with staff and patients for service improvements (47–49). It is an inclusive approach where end users and stakeholders work together to create a focused solution (46). Co-design could be a useful approach for hospital foodservices to consider when designing and executing a food and food-related waste audit as it targets many of the items discussed by participants in this study to drive audit completion, such as teamwork, communication, and engagement. For example, gaining buy-in from staff members was an important driver for audit related behaviors. Using a co-design process that involves key stakeholders involved in hospital food and food-related waste audits, such as academics, foodservice staff, managers, policymakers, and hospital executives, could: promote buy-in from executives who are required to approve audits occurring, allow managers to allocate staff members to audit specific roles without derailing everyday practice, empower foodservice staff to speak up and identify complications with suggested audit methods, and for all members involved to negotiate audit expectations. A co-design approach may also reduce preliminary audit errors and staff resistance, which participants from this study expressed concerns about. The research presented here builds on the first half of the co-design method (the generative research phase) (50) from *discovering* the current practice gap and *defining* the associated problems with the desired behavior. Future research will be able to close the second half of the co-design cycle (the developmental design phase) (50) by *developing* an appropriate intervention and executing its *delivery* based on the evidence.

Despite the aim of this research, some participants explained how they had previously completed successful surplus plating line waste or patient plate waste audits in their facilities, which is a somewhat common practice in hospital foodservices to understand forecasting numbers and patient nutrition intake (10). Although these past audits were not of aggregate food waste, and only focused on one section of foodservice, there is still value in completing imperfect audit practices. The outcomes from completing these audits provide rough calculations for waste amounts, upskill staff in auditing techniques, can generate interest toward the broader food and food-related waste issue in hospitals, and promote awareness of waste not being viewed as overproduction or unconsumed food, but as what it truly is, waste. The transition to completing aggregate food and food-related waste audits may be easier in facilities that have completed segmented audits previously, as stakeholders can recognize the similarities in practice, but on a larger scale. If altering practice to complete aggregate food and food-related waste audits rather than sectioned audits, it should be a permanent change in practice (i.e., once altered, foodservices should not revert back to previous practice), this would minimize any possible change fatigue in staff behaviors (51). Gathering information on the planning, execution, and data analysis phases from those involved post aggregate audit completion such as method feasibility, personal experiences, and staff fidelity to audit procedures can then provide insight to limitations and areas for improvement in future iterations.

A transformation in hospital policy regarding waste measurement is a further strategy beyond the identified intervention functions that can promote the transition to regular aggregate food and food-related waste audits. Different states and territories in Australia have diverse recommendations for the frequency and types of food waste measurement in hospital foodservices (52–54), but a national policy could promote standardized practice for all locations in the country, making widespread implementation easier. Currently, international policy is targeting the management of food waste in hospitals, mandating organizations to provide proof of food waste reduction and diversion from landfill techniques in their facilities, which could be another avenue for policy to promote food waste measurement (55–57). Additionally, based on the findings of this research, empowering foodservices staff (reflective motivation) with the opportunity to conduct an audit via allocating a nationally recognized day (58) or week (59) for standardized food waste measurement to occur, similar to the Nutrition Care Day Survey, may be a strategy to promote aggregate food and food-related waste audit completion and generate baseline data to improve. This would require audit resources such as labor and equipment (physical opportunity), and perhaps an identical training package to be delivered to participating sites to address education and skill requirements (physical and psychological capability), which could be provided by a governing body (e.g., Institute of Hospitality in Healthcare or Dietitians Australia) that engage with and promote quality improvement activities for the entire profession. Providing incentivization (as used by participants in this study) to well performed or the most accurate audits during this allocated time could reinforce hospitals to participate (automatic motivation), and provide an opportunity to support workforce interest through broadcasting their achievements via internal or external media. Moving forward, hospitals should address each COM-B construct and TDF domain through the use of a combination of multifaceted intervention functions to promote a higher chance of achieving long term aggregate audit completion.

### 4.1. Strengths and limitations

A strength of this research is the use of multiple published and validated (25) behavior change theories and frameworks (i.e., TDF, BCW, and COM-B) to support the rationale and findings. The method used to report on these is transparent and replicable, which may support future research to inform the development of other interventions targeted toward hospital foodservices. Although, due to questions in the interview schedule not being designed specifically around each TDF domain as done in some studies using this framework (60), questions may have been skewed to certain domains and therefore highlighting more dominant themes without intention. Considering this, and that there were a large number of non-respondents, there may be other experiences and perspectives to be explored. Nevertheless, a further strength to this study is the range of different hospital types and sizes providing a varied sample.

Some hospitals had completed plate waste audits previously and participants may have been more motivated to participate as they were aware of their own good practice. The oversampling of dietitians (35%) and managers/supervisors (50%) compared to workers at the coal face (10%) such as dishwashers, cooks, and foodservice assistants may have skewed results, but staff on the front line are a time-poor population and difficult to access for interviews. In addition, participants in managerial positions described their own perspectives of what barriers and enabler’s foodservice workers may experience when completing food and food-related waste audits compared to what those foodservice workers may actually experience. However, this does not disregard the findings from this research as those interviewed in this study have strong knowledge of foodservice operations, which were the targeted population.

## 5. Conclusion

This study used a systematic approach from behavioral science (COM-B/TDF/BCW) to further understand possible behavior change interventions that may enable aggregate food and food-related waste audits in hospital foodservices. The research findings add to the small body of evidence on behavior change in this unique population of hospital foodservice staff, highlight the perspectives of multiple stakeholders, and illustrate the importance of situational context that influences this complex practice gap. It is recommended that co-design methods combining key stakeholders be adopted, as the findings from this study suggest the composite nature of food and food-related waste audit design and eventual completion require new knowledge, repeated communication, upskilling, workplace maneuvering, staff engagement, and collaborative teamwork, which cannot be achieved alone. Addressing each behavioral construct and relevant domains during intervention development may increase the likelihood of effective and locally feasible solutions that could improve the implementation of aggregate food and food-related waste audits in hospital foodservices.

## Data availability statement

The datasets presented in this article are not readily available because data are not available for sharing as participant consent precludes this. Requests to access the datasets should be directed to jorja.collins@monash.edu.

## Ethics statement

The studies involving humans were approved by Monash University Human Research and Ethics Committee. The studies were conducted in accordance with the local legislation and institutional requirements. The participants provided their written informed consent to participate in this study.

## Author contributions

NC conducted the interviews, collated, analyzed and interpreted the data and wrote the manuscript. JC, JP, and DG supervised this process and critically reviewed the manuscript. NC, JC, JP, and DG contributed to the conceptualization and design of the study and have read and approved the final publication. All authors contributed to the article and approved the submitted version.

## Funding

NC received a departmental scholarship for his Ph.D. from Monash University’s Department of Nutrition, Dietetics and Food, and a King and Amy O’Malley Trust Scholarship during this study.

## Conflict of interest

The authors declare that the research was conducted in the absence of any commercial or financial relationships that could be construed as a potential conflict of interest.

## Publisher’s note

All claims expressed in this article are solely those of the authors and do not necessarily represent those of their affiliated organizations, or those of the publisher, the editors and the reviewers. Any product that may be evaluated in this article, or claim that may be made by its manufacturer, is not guaranteed or endorsed by the publisher.
